# Reliable but Not Interchangeable: What Validated Instruments Measure When They Grade an Anastomosis

**DOI:** 10.3390/jcm15155779

**Published:** 2026-07-23

**Authors:** Teona Z. Carciumaru, Victor Esanu, Clemens M. F. Dirven, Dalibor Vasilic, Victor Volovici

**Affiliations:** 1 Department of Plastic and Reconstructive Surgery, Erasmus MC University Medical Center, 3015 GD Rotterdam, The Netherlands; t.carciumaru@erasmusmc.nl (T.Z.C.); d.vasilic@erasmusmc.nl (D.V.); 2 Department of Neurosurgery, Erasmus MC University Medical Center, 3015 GD Rotterdam, The Netherlands; c.dirven@erasmusmc.nl; 3 Center for Complex Microvascular Surgery, Erasmus MC University Medical Center, 3015 GD Rotterdam, The Netherlands; v.esanu@erasmusmc.nl; 4 Department of Experimental Microsurgery, Simulation and Experiment Center, Iuliu Hațieganu University of Medicine and Pharmacy, 400347 Cluj-Napoca, Romania

**Keywords:** microsurgery, microsurgical training, surgical education, surgical assessment, competency-based training

## Abstract

**Background/Objectives**: Multiple validated microsurgical assessment instruments exist that differ in design and theoretical approach. Product-based tools evaluate the quality of the completed anastomosis, while process-based tools assess technique during task execution. Whether these tools measure the same underlying construct and can be used interchangeably has not been directly examined. This study compared the behaviour of three validated instruments, ALI, MARS10, and SMaRT, when applied to identical performances. **Methods**: Forty-five participants from 26 institutions across 14 countries performed an eight-stitch end-to-end vascular anastomosis on a chicken leg model, independently evaluated by two trained graders using all three instruments. Interrater reliability was assessed using intraclass correlation coefficients (ICC(2,1)), rank-order agreement using Spearman’s rho and Kendall’s W, and score distributions were examined for floor and ceiling effects. **Results**: All three instruments demonstrated high interrater reliability (ICC: ALI 0.955, MARS10 0.939, SMaRT 0.960), though product-based tools showed wider confidence intervals. Systematic rater differences were observed across all instruments. Rank correlations were strongest between ALI and MARS10 (ρ = 0.913), with moderate correlations between process-based and product-based tools (ρ = 0.698–0.749). MARS10 showed a ceiling effect, SMaRT underutilised its upper range, and ALI had the greatest score variability. **Conclusions**: Although demonstrating high interrater reliability, the instruments differ in rater variability, discriminatory capacity, and the aspects of performance prioritised, embodying different definitions of surgical skill. Current tools are not interchangeable, and instrument selection carries potential consequences for training and research. Future research in technology-driven approaches could offer a path toward more objective and consistent evaluation that is less dependent on human interpretation.

## 1. Introduction

Microsurgery is one of the most technically demanding areas of surgical practice, requiring a high degree of precision and refined motor control. The development of these skills is notoriously slow and challenging, with both steep and prolonged learning curves [1,2]. Accurate and meaningful assessment of surgical performance is central not only to training but to the clinical outcomes that follow [3,4,5]. Assessment tools inform feedback, guide progression, and ultimately support broader decisions about trainee development [6]. For these reasons, such tools must be reliable, consistent, interpretable, and capable of meaningfully distinguishing between levels of skill.

Several validated instruments have been developed for evaluating microsurgical performance, each reflecting a different conception of what technical skill means. Some prioritise the quality of the final product, whereas others emphasise the process by which the task is performed. The Microsurgical Anastomosis Rating Scale (MARS10) takes a product-based approach, assessing the completed anastomosis against structured criteria covering suturing technique, knot tying, and anastomosis closure [7]. The Anastomosis Lapse Index (ALI) similarly examines the end result, but through an error-based approach, identifying and quantifying technical mistakes in the final result [8]. In contrast, the Structured Assessment of Microsurgical Skills (SMaRT) scale evaluates performance during task execution, incorporating both procedural technique and outcome [9]. Although all three instruments target the same technical task, they do not necessarily measure the same underlying construct. Their design differences may translate into variation within ranking behaviour, discriminatory capacity, and limit interchangeability between instruments.

Despite the availability of multiple validated microsurgical assessment tools, their direct comparison is limited. In practice, institutions, training programs, and research groups adopt whichever assessment method suits their setting, implicitly assuming a degree of equivalence that has not been clearly established. Whether these assessment tools capture overlapping or distinct dimensions of skill is an open question. Product-based and process-based approaches may reflect fundamentally different perspectives on performance, rather than interchangeable measures of the same construct.

The primary aim of this observational methodological study is to compare the behaviour of three validated microsurgical assessment tools, namely ALI, MARS10, and SMaRT, when applied to the same set of performances. We examine interrater reliability, discriminatory capacity, ranking behaviour across tools, and assess the level of agreement between product-based and process-based evaluations. In doing so, we seek to clarify how differences in assessment tool design shape the interpretation of microsurgical performance and to inform the selection and development of evaluation tools in surgical training and research.

## 2. Materials and Methods

### 2.1. Study Design

The study was conducted using an existing dataset of 50 participants recruited at Erasmus MC University Medical Center Rotterdam, the Netherlands, and the University of Medicine and Pharmacy “Iuliu Haţieganu”, Cluj-Napoca, Romania. Data collection was carried out under institutional approval at both sites (MEC-2024-0656 Rotterdam; AVZ247/07.09.2022 Cluj-Napoca), and all participants provided informed consent. Participant trials were eligible for inclusion in the present analysis if complete operative video recordings and corresponding scoring data were available. Of the 50 participants, 5 were excluded due to incomplete data. Each participant contributed one performance, with 45 trials included. The cohort represented multiple institutions and a range of microsurgical experience levels.

The microsurgical task consisted of performing an 8-stitch end-to-end vascular anastomosis on a chicken leg model [10,11]. Although participants represented multiple institutions, all performed the same standardised task under comparable conditions in one of the two centers. The same researcher supervised all sessions. Procedures were recorded using the operating microscope video feed. Following task execution, the completed anastomoses were cut open and recorded from both the internal luminal and external surfaces to allow assessment of the end product.

The same two evaluators independently evaluated every performance once using each of the three validated microsurgical assessment tools: ALI, MARS10, and SMaRT. The ALI and MARS10 scales were applied during the end-product inspection, whereas the SMaRT scale was applied using both the end-product images and the procedural video recordings. All assessment tool structures may be found in Appendix A.

The ALI score quantifies technical errors in the completed anastomosis by identifying predefined error types, with scores accumulating such that higher values reflect poorer technical performance. Scores below 3 correspond to expert-level performance, scores between 3–6 to intermediate level, and scores above 6 to novice level. The MARS10 score assesses five domains covering suture placement, knot quality, and anastomotic closure, resulting in a total score from 0–10. The SMaRT score evaluates intraoperative microsurgical technique across nine performance domains, each rated on a 5-point Likert scale, for a total score ranging from 9–45. For both the MARS10 and SMaRT scores, higher scores reflect better technical skill.

Prior to formal grading, both graders participated in a calibration session to align their interpretation of scoring criteria and reduce variation in approach. All recordings were then scored independently, with graders blinded to participant information. No additional participant-level data were used at any stage of scoring.

During the preparation of this manuscript, the author(s) used ChatGPT-5.5 by OpenAI for the purposes of language editing. The authors have reviewed and edited the output and take full responsibility for the content of this publication.

### 2.2. Statistical Analysis

Interrater reliability for ALI, MARS10, and SMaRT was assessed using intraclass correlation coefficients (ICC), applying a two-way random-effects model with absolute agreement (ICC(2,1)) across both raters and all 45 participants. Scores were subsequently averaged across raters for each participant for each instrument. Systematic differences between raters were assessed using Wilcoxon signed-rank tests. In addition to the total-score reliability, interrater reliability was examined at the level of individual components, computing ICC(2,1) for each domain of MARS10 and SMaRT and for each predefined error category of ALI, to determine whether some components are scored more consistently than others.

Rank-order agreement between instruments was assessed using Spearman’s rho for pairwise associations, and Kendall’s W for overall concordance. ALI scores were reverse-coded to align directionality with MARS10 and SMaRT. Two-sided tests were used with significance set at *p* < 0.05.

Floor and ceiling effects were evaluated as the proportion of scores within the lower or upper 10% of each instrument’s observed range. This threshold was used to provide a consistent definition across scales with different structures. MARS10 and SMaRT have fixed limits, whereas ALI is an error-count score for which a theoretical range cannot be defined because errors may recur.

No a priori sample size or power calculation was performed because this was a secondary analysis of an existing dataset.

## 3. Results

A total of 45 participants were included in the study, representing 26 unique institutions across 14 countries (Table 1).

The ALI score is an error-based metric with a theoretical range from 0 to infinity, where lower values indicate better performance. The median error count was 9.5 [IQR 5.5–13.5], spanning an observed range of 0.5–27.5. In contrast, both MARS10 and SMaRT are bounded scales, ranging from 0–10 and 9–45 respectively, with higher scores indicating better performance. MARS10 yielded a median of 6.5 [IQR 4.0–8.0] across an observed range of 0–9.5, while SMaRT showed a median of 25.0 [IQR 18.5–30.0], with values spanning 9.0–38.5.

### 3.1. Interrater Reliability

Interrater reliability was high across all three assessment tools. ICC(2,1) values for the total scores were 0.955 (95% CI 0.503–0.987) for ALI, 0.939 (95% CI 0.797–0.975) for MARS10, and 0.960 (95% CI 0.925–0.978) for SMaRT, reflecting strong rater agreement in each case (Table 2). That said, the two product-based tools, ALI and MARS10, showed considerably wider confidence intervals than the process-based SMaRT scale for total score. The domain-level results also suggest that the instruments are less consistent when assessing fine-grained technique or multi-part qualitative behaviours.

Statistically significant systematic differences between raters were observed for all instruments (Table 3). Rater 1 assigned lower scores than Rater 2 for ALI, and higher scores for MARS10 and SMaRT. Given that lower ALI scores and higher MARS10 and SMaRT scores each indicate better performance, these differences were directionally consistent across tools. Variability between raters differed by instrument. ALI and MARS10 showed relatively narrow limits of agreement, indicating consistent scoring differences, whereas SMaRT demonstrated wider limits of agreement, reflecting greater variability in absolute scores between raters.

### 3.2. Rank Correlation

**Figure 1:**
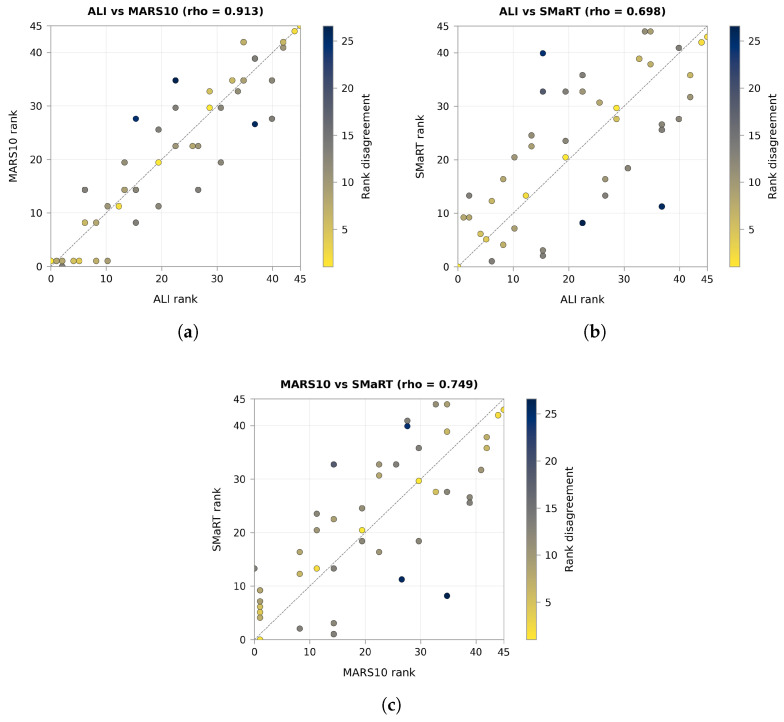
Pairwise rank correlations between microsurgical assessment tools: (**a**) ALI vs MARS10. (**b**) ALI vs SMaRT. (**c**) MARS10 vs SMaRT.

Participant rankings were strongly correlated across tools after reversing ALI to align directionality (Figure 1). The closest agreement was between ALI and MARS10 (ρ = 0.913, *p* < 0.001), with moderate correlations for ALI versus SMaRT (ρ = 0.698, *p* < 0.001) and MARS10 versus SMaRT (ρ = 0.749, *p* < 0.001). Overall concordance across all three tools was high (W = 0.855, $\chi^{2}$ = 112.82, *p* < 0.001), indicating broadly similar participant rankings regardless of instrument.

### 3.3. Score Distributions

ALI showed the greatest relative variability in scores, followed by MARS10 and SMaRT, indicating a broader spread of scores for ALI and comparatively less dispersion for SMaRT (Table 4). MARS10 demonstrated a notable ceiling effect, whereas this was minimal for ALI and SMaRT. Floor effects were low overall, though slightly more pronounced for ALI.

**Figure 2:**
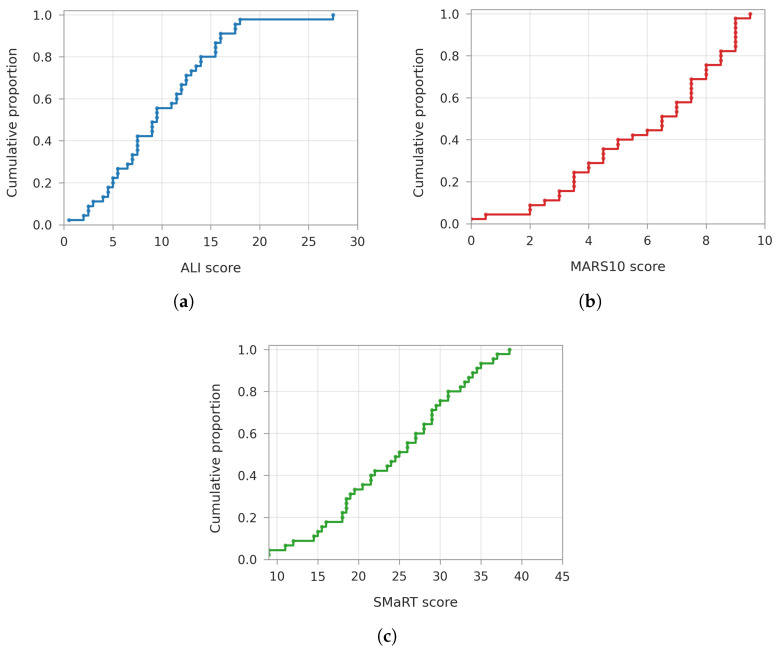
Empirical cumulative distribution functions (ECDF) of microsurgical assessment scores: (**a**) ALI. (**b**) MARS10. (**c**) SMaRT.

In terms of score utilisation, MARS10 spanned nearly its full range, while SMaRT did not reach its upper bound (maximum 38.5/45), suggesting underutilisation of higher scores in this cohort. ALI scores ranged from 0.5 to 27.5 with limited clustering at higher values. Taken together, MARS10 exhibited a ceiling effect, SMaRT showed underutilisation of the upper range, and ALI demonstrated greater variability with mild clustering at lower error counts. These distributional differences are visually illustrated in Figure 2.

## 4. Discussion

This study compared how three validated microsurgical assessment tools, ALI, MARS10, and SMaRT, behave when applied to identical microsurgical performances. All three instruments demonstrated high interrater reliability, but systematic rater differences, distinct score distributions, and only moderate correlations between process- and product-based measures indicate that they are not interchangeable and do not assess microsurgical skill in the same way. Each tool carries its own constraints, both in design and in how raters interpret it. High agreement on a narrow or incomplete definition of skill does not make that definition correct; it simply reproduces it more consistently.

A key finding is that high interrater reliability coexisted alongside systematic rater bias. The consistent direction of these differences across tools suggests that raters applied the criteria consistently but used different internal benchmarks. The limits of agreement further showed that the same performance could receive meaningfully different scores, particularly with the process-based SMaRT scale. For ALI, the limits ranged from −3.32 to 0.74, crossing the established thresholds of below 3 for expert performance and above 6 for novice performance [8]. Participants scoring near these boundaries could therefore be classified differently depending on the rater, with potential consequences for competency-based training and progression decisions.

SMaRT produced the most precise ICC estimates, suggesting more consistent differentiation between participants despite greater variability in absolute scores. The product-based assessments had wider confidence intervals. This distinction is important because consistent ranking does not necessarily imply agreement in absolute scores. Process-based assessment may provide more reproducible comparative evaluations, but remains more open to interpretation. Although ALI and MARS10 use more constrained criteria and showed more consistent rater differences, all three tools ultimately depend on human judgement to interpret criteria, identify errors, and assign scores. This subjectivity is also unevenly distributed within each instrument. Sub-domains such as knot quality require more interpretation than countable features such as stitch number, meaning that a single global reliability estimate may conceal substantial item-level variation.

Although all tools assess the same task, rank correlations between them were only moderate. The strongest agreement was found between ALI and MARS10, both of which focus on the end product and therefore capture overlapping aspects of technical performance. Their weaker correlations with SMaRT suggest that process-based evaluation captures additional dimensions, including efficiency, instrument handling, and procedural flow. This supports the view that microsurgical skill is multidimensional, and that product-based and process-based assessment tools offer complementary rather than interchangeable perspectives. More fundamentally, however, none of the instruments are anchored to an external gold standard. Each has been validated mainly against expert judgement or other rating scales, so scores are calibrated relative to one another rather than to the outcome that ultimately matters: vessel behaviour once blood flow is restored.

Differences in score distribution patterns further reflect the influence of scale design. MARS10 exhibited a ceiling effect, limiting discrimination among higher-performing participants and suggesting it is more suitable for early-stage training, in line with findings reported in its original validation study [7]. ALI showed greater overall variability but with clustering at lower error counts, indicating sensitivity to performance differences at intermediate levels but reduced resolution among more skilled participants. SMaRT had a broader distribution without ceiling clustering but underutilised its upper range, also suggesting incomplete capture of high-end performance. This wider spread may reflect its multidimensional, process-based design, which also incorporates elements of the final outcome. Overall, each scale’s structure shapes its discriminatory capacity and the aspects of performance it captures.

Instrument selection should therefore be guided by the intended assessment purpose rather than reliability alone. This study cannot determine which instrument should be preferred in specific educational or research settings because educational and clinical outcomes were not evaluated. It does, however, identify relevant differences: MARS10 showed a ceiling effect, ALI the greatest score variability, and SMaRT assessed procedural as well as final-product performance (Table 5). These findings may inform selection, but comparative suitability requires further study.

Several limitations should be considered. The study did not include an external reference standard such as anastomotic patency or clinical outcome. We therefore cannot determine which instrument most accurately reflects clinically meaningful performance; our conclusions are limited to the relative behaviour and interchangeability of the three instruments. Additionally, only two raters evaluated the performances, and both participated in a calibration session. Although calibration standardised application of the scoring criteria, it may also have increased agreement compared with routine educational settings involving larger and more heterogeneous groups of assessors. Another limitation is the absence of a universally accepted gold standard for objective assessment in microsurgery, which prevents direct comparison against a single reference method and limits interpretation to comparisons between existing validated instruments. The floor and ceiling thresholds were defined using the lower and upper 10% of the observed score range, which avoids imposing an arbitrary cutoff on the unbounded ALI but makes these estimates sample-dependent. Finally, performance in a simulated setting may not fully reflect the complexity of clinical practice [12].

These findings carry practical implications for microsurgical education. Product- and process-based assessments should not be treated as interchangeable, and future frameworks should examine whether combining complementary dimensions of performance improves discrimination, feedback, and prediction of competence. The persistent influence of rater interpretation also supports further development of more objective, technology-assisted methods.

Sensor-based and automated metrics offer an interesting future perspective that could quantify aspects of performance such as motion efficiency, instrument handling, and procedural flow with greater precision and consistency than human rating alone [13,14,15]. Future studies should determine which metrics are most informative, how they should be weighted, and whether they can reduce trainer burden while supporting longitudinal assessment of skill acquisition and maintenance. Future research could also explore how machine-learning-based assessment models should be structured. They should not simply reproduce human-defined scores, as this risks preserving the same circularity in which subjective criteria are treated as ground truth and then applied more consistently by an algorithm. Models should make explicit which aspects of performance they are designed to capture and be validated against clinically meaningful outcomes.

At a deeper level, this work invites reflection on what should be expected from a surgical assessment system. Such systems are not neutral measurement tools: their structure reflects which aspects of performance a field chooses to value and, therefore, how skill is defined. Their value lies not only in producing reproducible scores, but also in characterising performance in a way that is fair, discriminative, clinically relevant, and minimally dependent on individual evaluator interpretation. Future frameworks should capture the multidimensional nature of microsurgical skill, balance technical outcome with procedural execution, and remain practical, interpretable, and useful for longitudinal training. Ultimately, reliability alone is insufficient, and construct validity must not be forgotten. Circularity arises when instruments are validated primarily against expert judgement or one another, allowing consistency within existing definitions of skill to be mistaken for validity. ICC, limits of agreement, and rank correlations assess different properties; none alone establishes validity or predicts educational or clinical outcomes. Future research should therefore identify which measurable features of performance are most closely associated with meaningful surgical outcomes, extending assessment beyond what a grader can observe on a bench model.

## 5. Conclusions

ALI, MARS10, and SMaRT all demonstrated high interrater reliability when applied by two calibrated expert raters. However, they differ in how they score performance, the variability they introduce, and the dimensions of skill they capture. These differences stem from underlying variation in construct and design and demonstrate that current tools are not interchangeable. Future research should evaluate more comprehensive and objective approaches that integrate multiple dimensions of performance and explore whether technology-assisted methods can reduce dependence on subjective interpretation.

## Figures and Tables

**Table 1:** Participant characteristics.

Characteristic	Category	Value
Age, mean ± SD (years)	–	31.8 ± 9.7 (20–54)
Sex, *n* (%)	Female	19 (42.2)
	Male	26 (57.8)
Status, *n* (%)	Student	15 (33.3)
	Researcher	3 (6.7)
	Resident	11 (24.4)
	Consultant	15 (33.3)
	Surgical trainer	1 (2.2)
Specialty, *n*	Neurosurgery	21
	Plastic surgery	2
	General surgery	1
	Obstetrics & gynaecology	1
	Radiology	1
	None	19

**Table 2:** Item-level interrater reliability by assessment domain. ICC(2,1) and 95% confidence intervals are computed per component. Higher values indicate more consistent scoring between raters.

Instrument and Domains	ICC(2,1)	95% CI
**ALI**		
Anastomosis line disruption	0.899	0.824–0.943
Backwall/sidewall stitch	0.885	0.801–0.935
Oblique stitch	0.880	0.791–0.932
Wide bite	0.771	0.621–0.867
Partial-thickness stitch	0.851	0.717–0.920
Unequal suture spacing	0.839	0.724–0.908
Vessel-wall tear	1.000	1.000–1.000
Overly tight suture	0.836	0.465–0.934
Intraluminal threads	0.835	0.719–0.905
Edge overlap	0.740	0.484–0.865
Total score	0.955	0.503–0.987
**MARS10**		
Perpendicular stitches; correct bite size	0.846	0.736–0.912
Symmetry; parallelism; correct spacing; correct number of sutures	0.656	0.454–0.795
Cut ends of proper length; square knots	0.647	0.439–0.790
Appropriate vessel edge approximation; appropriate suture tightness	0.781	0.619–0.876
Leakproof and tight closure; non-obstructed lumen; no backwall stitches; all suture ends extraluminal	0.926	0.869–0.958
Total score	0.939	0.797–0.975
**SMaRT**		
Instrument handling	0.779	0.419–0.901
Respect for tissue	0.797	0.520–0.904
Efficiency	0.768	0.560–0.876
Suture handling	0.629	0.412–0.778
Suturing technique	0.645	0.436–0.788
Quality of knot	0.692	0.503–0.818
Final product	0.852	0.745–0.916
Operation flow	0.775	0.618–0.872
Overall performance	0.904	0.821–0.948
Total score	0.960	0.925–0.978

**Table 3:** Interrater bias and limits of agreement across assessment tools.

System	Mean Difference (R1–R2) ^1^ (SD)	Limits of Agreement	*p* (Wilcoxon)
All	−1.29 (1.04)	−3.32–0.74	<0.001
MARS10	0.53 (0.76)	−0.95–2.02	<0.001
SMaRT	0.69 (2.17)	−3.57–4.95	0.037

^1^ R1: rater 1; R2: rater 2.

**Table 4:** Distribution characteristics of microsurgical assessment scores.

System	Mean (SD)	Observed Range	Coefficient of Variation (%)	Floor Effect (%)	Ceiling Effect (%)
All	9.9 (5.4)	0.5–27.5	54.4	11.1	2.2
MARS10	6.0 (2.6)	0–9.5	42.9	4.4	17.8
SMaRT	24.4 (7.8)	9.0–38.5	32.1	6.7	6.7

**Table 5:** Practical characteristics and potential applications of the assessment instruments based on findings from the present cohort.

Instrument	Focus	Limitations Observed in This Study	Potential Applications
ALI	Errors in the completed anastomosis	Greatest score variability; rater differences near proficiency thresholds; reduced discrimination at low error counts	Targeted identification and quantification of end-product technical errors; useful for structured formative feedback
MARS10	Structured quality assessment of the completed anastomosis	Ceiling effect; weaker reliability in some domains	Rapid, simple assessment of completed anastomosis quality, particularly in basic training
SMaRT	Procedural performance and final product	Requires live or recorded procedures; upper range underused; greater variation in absolute scores between raters	Procedural coaching and assessment where the quality of task execution is of primary interest

## Data Availability

The dataset presented in this article is on not readily available as the data is part of an ongoing study, but would be shared upon reasonable request to the senior author.

## Abbreviations

The following abbreviations are used in this manuscript:

ALI	Anastomosis Lapse Index
MARS10	Microsurgical Anastomosis Rating Scale
SMaRT	Structured Assessment of Microsurgical Skills
ICC	Intraclass correlation coefficient
CI	Confidence interval
SD	Standard deviation
IQR	Interquartile range
ECDF	Empirical cumulative distribution function

## Appendix A. Assessment Tool Structure

### Appendix A.1. ALI [8]

**Table A1:** ALI error types and definitions. Each error type is scored as a count from 0 upward, with higher scores indicating more errors.

Error Type	Definition	Score Range
Error 1	Disruption of the anastomosis line created by the opposed vessel ends.	0+
Error 2	Inadvertently catching the back wall or side wall when taking suture bites, causing occlusion of the lumen.	0+
Error 3	Placing an oblique stitch that causes tissue distortion.	0+
Error 4	Taking too wide a bite, resulting in excess tissue inclusion.	0+
Error 5	Placing a stitch that does not go through the full thickness of the vessel wall, allowing some of the intimal layer to conceal part of the stitch.	0+
Error 6	Unequal spacing of sutures, with sutures placed parallel to one another rather than twice the expected spacing.	0+
Error 7	Causing a visible tear in the vessel wall.	0+
Error 8	Placing an excessively tight suture that strangulates the tissue.	0+
Error 9	Leaving suture threads within the vessel lumen.	0+
Error 10	Allowing excessive edge overlap at the anastomosis.	0+

### Appendix A.2. MARS10 [7]

**Table A2:** MARS10 scoring criteria. Each criterion is scored from 0 to 2, with higher scores indicating better performance. The total score ranges from 0 to 10.

Domain/Criterion	0	1	2
Sutures: Bite orientation and size	Sutures not perpendicular to the anastomosis; wrong bite sizes	Intermediate performance	Sutures perpendicular; equal, proper bites
Sutures: Suture spacing	Asymmetrical sutures; poor suture spacing	Intermediate performance	Symmetrical, parallel sutures; appropriate suture number and spacing
Knot quality: Cut ends and knot shape	Cut ends too short or too long; knot not square	Intermediate performance	Cut ends of proper length; square knot
Knot quality: Tightness	Inversion or eversion of vessel edges; stitches too loose or too tight	Intermediate performance	Appropriate tightness
Anastomosis closure	Non-leakproof or obstructed anastomosis; back wall stitch; suture ends intraluminal	Intermediate performance	Leakproof, tight closure; non-obstructed anastomosis; all suture ends extraluminal

### Appendix A.3. SMaRT [9]

**Table A3:** SMaRT scoring criteria. Each criterion is scored from 1 to 5, with higher scores indicating better performance. The total score ranges from 9 to 45.

Domain	1	3	5
Instrument Handling	Repeated tentative or awkward moves; inappropriate use of instruments.	Competent use of instruments; occasionally stiff or awkward.	Fluid, concise moves with appropriate instruments.
Respect for Tissue	Frequent unnecessary tissue force or damage to vessels.	Careful tissue handling; occasional inadvertent damage.	Consistently handles tissue carefully and appropriately; minimal tissue damage.
Efficiency	Many wasted moves; regrasps multiple times; does not pull needle out of field.	Some wasted moves; regrasps occasionally; sometimes pulls needle out of field.	No wasted moves; grasps once only; always pulls needle out of field.
Suture Handling	Multiple passes; grasps tip of needle; pulls needle out not on the curve.	A few passes; sometimes grasps the tip; sometimes pulls needle out on the curve.	Sutures were consistently handled delicately under control; single passes; never grasps the tip; always pulls needle out on the curve.
Suturing Technique	Inaccurate needle placement; pulls needle through roughly; inefficient knot tying; too much movement of anastomosis/tissue with tying.	Inconsistent needle placement; rough/inconsistent needle passage; knot tying too loose, tight, or inefficient.	Accurate needle placement; takes needle out on curve; efficient tying; tissue/tissue stays still with tying.
Quality of Knot	Not square; loose; cut ends too long/short.	Partially square; somewhat loose; cut ends OK length.	Square, snug; cut ends proper length.
Final Product	Rough outer appearance; poor suture spacing; suture ends intraluminal.	Anastomosis inconsistently bunched or inverted; suture spacing inconsistent.	Smooth outer appearance; all suture ends extraluminal; appropriate spacing.
Operation Flow	Frequent lack of forward progression; stopped operating frequently and seemed unsure of next move.	Demonstrates some forward planning with reasonable progression of procedure.	Proceeds at appropriate pace with obviously planned course.
Overall Performance	Unable to perform the procedure.	Able to perform the procedure.	Mastery of all aspects.

Scores of 2 and 4 represent intermediate performance between the adjacent anchored descriptions.
